# Treatment of Diphtheria

**Published:** 1891-01-24

**Authors:** 


### The Practitioner's Mirror and Retrospect.

[*The Editor will be glad to receive offers of co-operation and contributions from members of the profession. There is no doubt that much valuable material full of interest and instruction is at present lost to science. This is largely so in the case of* (1) *the younger and less-known members of the hospital staff* (2) *those connected with cottage hospitals, and* (3) *the great body of medical practitioners not attached to any institution. The co-operation of all is cordially invited to enable the Editor to make* The Hospital *of the utmost possible use and value to the profession. Specialists willing to review books on their special subjects are invited to communicate this fact at once. All letters should be addressed to* The Editor, The Lodge, Porchester Square, London, W.]

### MEDICINE.

### TREATMENT OF DIPHTHERIA.

Dr. Hermann Wolff, of Freiburg, i. B., having for many years treated every case of diphtheria by spraying so long as it was possible to check the progress of the local manifestations, and having used Liq. Ferri Perchlr. Liq. Sod. Chlorinatæ, Carbolic acid, Quinine, and Iodine water, has latterly discarded them all in favour of the "etherial" oils. He has tried turpentine, oil of peppermint, &c., but prefers menthol in the form of a fine powder with sugar in the proportion of 1 : 10 or 1 : 20. He applies it himself once daily, instructing the nurse to use it twice or thrice each day. The application is made with a large soft camel's hair brush, and if, as often happens, portions of the membrane adhere to the brush it is dipped in water, wiped, and the powder reapplied. On the second or third day the membrane falls off, leaving a clean raw surface with here and there small ulcerous depressions. He then dusts the surface again with the powder either by means of the brush or of an insufflator. Internally he uses antipyretics, preferring antifebrin. He also approves and makes use of Rosenbach's menthol inhalations, employing for this purpose Weigl's gasometer apparatus, passing the air through cotton-wool saturated with menthol, in a cylinder interposed between the gasometer and the inhaler. This procedure he finds of remarkable benefit in the treatment of tuberculosis while the patient enjoys the cool sensation of the inhalation which, with this apparatus, penetrates the smallest ramifications of the air passages.

